# Research on optimization of spray drying conditions, characteristics of anthocyanins extracted from *Hibiscus sabdariffa* L. flower, and application to marshmallows

**DOI:** 10.1002/fsn3.3898

**Published:** 2024-01-09

**Authors:** Nhon Thi Ngoc Hoang, Nga Ngoc Kieu Nguyen, Ly Thi Kim Nguyen, Anh Thi Hong Le, Dao Thi Anh Dong

**Affiliations:** ^1^ Faculty of Food Science and Technology Ho Chi Minh City University of Industry and Trade (HUIT) Ho Chi Minh City Vietnam; ^2^ Department of Food Technology, Faculty of Chemical Engineering Ho Chi Minh City University of Technology (HCMUT) Ho Chi Minh City Vietnam; ^3^ Vietnam National University Ho Chi Minh City (VNU‐HCM) Ho Chi Minh City Vietnam

**Keywords:** anthocyanin, antioxidant capacity, *Hibiscus sabdariffa* L., marshmallows, spray‐drying

## Abstract

Anthocyanin, a main‐colored bioactive compound found in *Hibiscus sabdariffa* L., is well‐known for a varied range of applications as food additives in foodstuff, and natural colorants in food, pharmaceutical, and printing industries. The study aimed to find out the suitable conditions for the spray‐drying process to obtain anthocyanin powder from the extract as well as characterized the powder. In addition, the obtained powder was applied to marshmallows and determined the acceptability of appearance, quality, and scavenging capacity of the candy. The carrier of maltodextrin and gum arabic was selected for spray‐drying, which had optimal conditions at 144°C and 7 mL/min, resulting in 100.22 mg/g anthocyanin content with an encapsulation efficiency of 93.87%. The obtained anthocyanin has appropriate moisture of 5.14%, quite appropriate bulk density, and tapped density, it also was high solubility, and poor flowability but easy compression. The shape of the particle by SEM analysis was low particle size (2–10 μm), wrinkled, unequal spherical size, rough surfaces with indentations, and slight cracks. The X‐ray diffraction (XRD) spectrum of the sample had very low crystallinity and diffuse wide peaks revealing that anthocyanin still exists inside maltodextrin particles. The FT‐IR spectrum had oscillations of characteristic groups of anthocyanin structure. Marshmallow samples added 5% anthocyanin powder gained high acceptability of appearance and maintained the scavenging capacity (DPPH) with an IC50 value of 7368.31 ppm after a month of storage.

## Graphical abstract

The optimal spray‐drying conditions to obtain the anthocyanin powder from *Hibiscus sabdariffa* L. extract with carrier agents of gum arabic and maltodextrin were determined. The anthocyanin pigment powder had acceptable hygroscopicity, high solubility, poor flowability, easy compression, and good encapsulation efficiency. Marshmallow added 5% anthocyanin powder gained high acceptability of appearance and maintained the scavenging capacity (DPPH) after a month of storage.

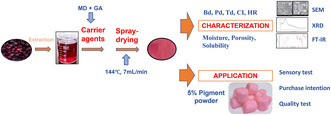

## 1 INTRODUCTION

Anthocyanins, water‐soluble organic pigment from a variety of plants, have been used in many fields due to their positive impact on human health. Their molecules are polar with hydroxyl, carboxyl, methoxyl, and glycolyl groups associated with the aromatic ring (Eichhorn & Winterhalter, 2005; Macz‐Pop et al., 2006). No adverse effects from anthocyanin derivatives were reported, even after ingestion of very high doses (Bertoia et al., 2016). Daily diets containing anthocyanins have gained a significant concern among the community because of certain effects, including anti‐inflammatory, anti‐diabetes, anti‐cardiovascular diseases, and antioxidant activities (de Pascual‐Teresa et al., 2010; Fairlie‐Jones et al., 2017; García‐Conesa et al., 2018). Thus, there is a growing demand to earn more sources of anthocyanins as well as fulfill available extraction processes to meet the practically increasing requirements. In addition, commercial applications of anthocyanins as colorants and pharmaceuticals require a convenient form such as a powder that is easy to use and store.

*Hibiscus subdariffa* L. (*H. subdariffa* L.), native to Asia or Tropical Africa, is well known for its high anthocyanin content. Its parts were used in traditional culinary, herbal, or fermented drinks, beverages, jelly, ice cream, wine, jam, puddings, and cakes. It has been widely used in local medicines to treat many health problems (Da‐Costa‐Rocha et al., 2014). Thus, there is a requirement to have a protocol to obtain anthocyanins from this plant and convert it to powder form for easy practical usage.

In previous research, we investigated the methods to improve the effectiveness of the anthocyanin extraction process (Nhon et al., 2022). In this study, the conditions for spray‐drying to have anthocyanin powder and its characteristics were examined. In addition, the initial application of the obtained pigment power in marshmallows and the acceptance and quality were also inspected.

## 2 MATERIALS AND METHODS

### 2.1 Materials

Calyxes of *H. sabdariffa* L. grown under GAP conditions (Ea Kar town, Ea Kar district, DakLak province, Vietnam). After collecting, the fresh calyxes were washed to remove the impurities, dried at 60°C until lower than 10% moisture content, grounded, and sieved through a 0.3 mm sieve, and preserved at 4°C in a zipper bag for experiments.

#### 2.1.1 Sucrose

Commercial grade granulated sugar cane, gelatin powder, glucose syrup, synthetic color (Carmine‐E120), and sodium chloride (food grade) were bought from supermarkets in Ho Chi Minh City, Vietnam.

#### 2.1.2 Chemicals

Maltodextrin (MD) 12DE (Sigma‐Aldrich), Gelatin (Ge) (Sigma‐Aldrich), Gum arabic (GA; Sigma‐Aldrich), enzyme cellulast 1.5 L (Novozyme). All chemicals and reagents were of analytical grade.

### 2.2 Methods

#### 2.2.1 Preparation of anthocyanin extract and spray drying

The conditions for anthocyanin extraction from *H. sabdariffa* L. were from previous studies (Nhon et al., 2022). Briefly, the material was treated with enzyme cellulase (2% based on the dry matter) for 1.5 h before microwave‐assisted extraction with power 450 W/g in 90 s. After the extraction, the mixtures were centrifuged to remove the residue and obtained the supernatant.

The carrier agents were selected to investigate namely maltodextrin (MD), maltodextrin (MD) + gelatin (Ge; MD:Ge, 1:1 w/w), and maltodextrin + gum arabic (GA; MD:GA, 1:1, w/w). Lab‐scaled spray dryer SD06 Labplant (UK) was used for spray drying. The suitable carrier was chosen for further investigation with the inlet air temperature (varied from 100 to 160°C), and feed flow rate (from 5 to 11 mL/min) according to experimental single‐factor designs. After the completion of these experiments, the RSM method was applied to find out the optimal conditions for spray drying anthocyanin. Then, the obtained anthocyanin powders were packed in an airtight sealed plastic packet and stored in a desiccator for further analysis.

##### Optimal experimental design

The response surface methodology (RSM) is used to optimize multiple variables simultaneously to achieve the desired response variables. RSM was used to derive the optimum process conditions using a two‐parameter central composite design (CCD) with 12 experimental runs including four replicates at the center point. The independent variables affecting the quality of the anthocyanin powder were the different levels of inlet air temperature and feed flow rate. Response variables such as yield recovery and anthocyanin content were evaluated. A second‐order polynomial equation was used to establish a link between the response variable (*Y*) and independent variables (*X*i).

$$Y=b_{0}+b_{1}X_{1}+b_{2}X_{2}+b_{1}b_{2}X_{1}X_{2}+b_{11}{X_{1}}^{2}+b_{22}{X_{2}}^{2}$$

#### 2.2.2 Determination of anthocyanin powder properties

##### Bulk density (Bd)

The bulk density of anthocyanin powder was determined manually by pouring 2 g of samples into a 10 mL graduated measuring glass cylinder. The bulk density of anthocyanin powder (g/cm^3^) was the ratio of the mass and the occupied volume of powder (Saifullah et al., 2016).

##### Particle density (Pd)

One gram of anthocyanin powder was poured into a 10 mL measuring cylinder with a glass stopper, then added 5 mL of petroleum ether and shaken for about 30 s so that all the particles were suspended. Next, 2 mL of petroleum ether was used to resin the wall of the cylinder and the total volume of the petroleum ether and suspended particles were read. The powder density was calculated as a ratio of powder mass to the total volume of petroleum ether and suspended particles (7 mL; Santhalakshmy et al., 2015).

##### Tapped density (Td)

The tapped density of the anthocyanin powder was measured by transferring a 2.5 g powder sample to a 10 mL graduated measuring glass cylinder. The tapped volume was measured after the sample was slowly dropped 100 times on a rubber carpet from a height of 15 cm. Later, we calculated the tapped density by dividing the weight of the powder by the tapped volume (Saifullah et al., 2016).

##### Solubility (S)

A total of 1 g of powder sample was mixed into 100 mL of distilled water in a magnetic stirrer (500 rpm for 30 min). The solution was transferred to experimental tubes and centrifuged at 2214 g (Model: Z 206A, Hermle, Germany) for 5 min. The supernatant was dried immediately in the oven‐dryer at 105°C for 5 h. The solubility (%) was calculated as the weight difference between the initial and the later one (Santhalakshmy et al., 2015).

##### Flowability (F)

The Carr Index (CI) and the Hausner Ratio (HR) were used to investigate the flow property of the samples, which were calculated from the bulk density (Bd) and tapped density (Td; Saifullah et al., 2016).

(1) $$\mathrm{CI}\;\left(\%\right)=\frac{\mathrm{Td}–\mathrm{Bd}}{\mathrm{Td}}\times 100$$

(2) $$\mathrm{HR}=\frac{\mathrm{Td}}{\mathrm{Bd}}\times 100$$

##### Porosity

The porosity (P) of the anthocyanin powder was calculated using particle density (Pd) and tapped density (Td) (Santhalakshmy et al., 2015).

(3) $$P\;\left(\%\right)=\frac{\mathrm{Pd}–\mathrm{Td}}{\mathrm{Pd}}\times 100$$

##### Water activity (Aw)

The water activity of the samples was determined using a digital water activity meter (Model 3TE, Aqualab). Triplet samples were analyzed, and the mean was obtained.

##### Encapsulation efficiency (EE)

The encapsulation efficiency was determined via total anthocyanin in total anthocyanin content (TAC) and surface anthocyanins content (SAC) of the microcapsules according to the description of Mahdavi et al. (2016).

(4) $$\mathrm{EE}\;\left(\%\right)=\frac{\mathrm{TAC}-\mathrm{SAC}}{\mathrm{TAC}}\times 100$$

#### 2.2.3 Structural and morphological analysis

##### Fourier transforms infrared spectroscopy

Fourier transforms infrared spectroscopy (FT‐IR) analysis was measured via infrared spectrometer (Tensor 37, Brucker), recording the spectrum between 4000/cm and 450/cm, with a resolution of 1/cm and accumulation of 100 scans (scan rate 0.5/cm/s). Potassium bromide was used in the blank sample (Joshi, 2012).

##### Scanning electron microscope

The particle morphology of the obtained anthocyanin powders was analyzed by scanning electron microscopy (SEM, JEOL). First, the samples were separately mounted on double adhesive tape stuck on gold aluminum pins. Finally, the individual coated sample was positioned in the SEM machine at an acceleration potential of 2.5 kV to observe the morphology.

##### X‐Ray diffraction

The obtained anthocyanin powders were identified by X‐ray diffraction (XRD) analysis using an X‐ray diffractometer (D2 Phaser, Bruker). The crystallinity of anthocyanin spray‐drying powder is evaluated by a scanning angle ranging from 30° to 40°. The scanning mode of CuKα radiation nickel filtering with an electrical pressure of 40 kV at a current of 0.8 mA, and a scanning speed of 4° per minute.

#### 2.2.4 Preparation of marshmallow

The main structure of the marshmallow is a gelatin matrix. Marshmallow samples were prepared according to the recipe and description of Dalia El‐Messiry et al. (2021) with some modifications. Gelatin (10.30% by mass of candy) was initially soaked in warm water of about 50°C (ratio of gelatin:water = 1:3) stirred vigorously with a mixer and cooked at 90°C to form a homogeneous gelatin solution. Continue to mix the gelatin solution with the mixture of the sugar (20% of total mass), glucose syrup 32DE (20% of total), sodium chloride food grade (0.13%), and the remaining water were manually homogenized and heated up to approximately 100°C. Hydrated gelatin was added and homogenized. The final solution remained on heating until 68–75°Bx. The mass was cooled to 80°C by manual stirring before adding orange flour (1%). The control sample was coloring by Carmine – a synthetic color at 0.01 g/100 g. Different levels of the anthocyanin powder (1%, 3%, 5%, and 7% of the marshmallow) were additionally mixed into the standard marshmallow. The final mixture was then poured into a cylindrical form with a 1 cm diameter, cooled overnight, and then cut to desired shape with around 1 cm in pieces. Candy samples were kept in sealed polypropylene plastic bags and analyzed after 0, and 30 days.

#### 2.2.5 Proximate analysis

The proximate analysis included moisture content, ash, protein, fat, reducing sugar, and total carbohydrates were determined according to the AOAC.

##### Moisture content

The moisture content in the sample was measured by using an oven drying method at 105°C until the constant weight according to AOAC Official Method 934.06.

##### Ash content

Ash content was determined using AOAC 942.05.

##### Protein content

The Kjeldahl method (AOAC 955.04) was used to determine the protein content of the sample.

##### Fat content

Fat content was determined by using the AOAC 985.15.

##### Fiber content

The determination of fiber content was based on the method AOAC 992.16.

##### Reducing sugar content

The content of reduced sugar was determined by AOAC 920.183.

##### Total carbohydrate content

The total carbohydrate content of the sample was calculated by the difference between the total weight of the sample (100%) and the summation of all other constituents (crude protein, fat, moisture content, ash, and crude fiber; Sun et al., 2014).

All proximate analysis was performed in triplicate, and an average reading was determined with standard deviation.

#### 2.2.6 Microbiological tests

The aerobic colony counts in candy samples were determined by AOAC Official Method 990.12. Enumeration of *Escherichia coli* and Coliform were determined by AOAC 991.14 and AOAC 998.08. AOAC Official Method 2018.02–2018 Enumeration of Yeast and Mold in Select Foods. *Clostridium perfringens* was determined by AOAC Official Method 974.38.

#### 2.2.7 Sensory acceptability

The acceptability test was conducted according to Lawless and Heymann (2010) and Montes Villanueva and Trindade (2010). For this evaluation, 110 marshmallow consumers were recruited among students and employees with no gender and age restrictions. The tests were conducted in private tasting booths. Each taster was guided about the use of the scale, the products, and how to evaluate the samples. Marshmallow samples were placed in white plastic plates covered with plastic film and served under white light, in disposable white plastic plates coded randomly with a three‐digit code number. The samples were also evaluated for appearance acceptability by using the 9‐point hedonic scale (9 = like extremely, 5 = neither like nor dislike, and 1 = dislike extremely). Then tasters were asked to range the samples in order of preference. Consumers were also required to fill out a questionnaire containing general questions concerning their marshmallow consumption habits. Finally, their purchase intention scale about tested products was asked, using a 5‐point structured scale (1 = certainly will not buy, 5 = certainly will buy).

#### 2.2.8 Antioxidant study

The radical‐scavenging activity (DPPH) was evaluated following the descriptions of (Tan et al., 2014). In short, the sample (0.6 mL) was added to DPPH solution (0.4 mL) and incubated in the dark for 30 min at room temperature. For the control, the DPPH solution was mixed with distilled water and a sample blank was prepared by replacing the DPPH with ethanol. The absorbance was measured via a UV–VIS spectrophotometer (at 525 nm). A low absorbance indicates a high free radical scavenging activity. The percent of DPPH‐scavenging activity was calculated by the following equation:

$$\text{DPPH scavening}\;\left(\%\right)=\left(1-\frac{A_{\text{sample}}-A_{\text{sample control}}}{A_{\text{blank}}}\right)\times 100$$

#### 2.2.9 Anthocyanin content determination

The anthocyanin content was spectrographically measured via spectrophotometer UV–VIS at 520 nm, and the content (mg/g of dry matter) was calculated by the following equation (Maier et al., 2008):

$$\text{Total anthocyanin}\;\left(\mathrm{mg}/g\right)=\frac{A\times D_{f}\times M_{w}}{\varepsilon \times L}$$

*A*=(*A*_λmax_ ‐ *A*_λ700_)_pH=1_ ‐ (*A*_λmax_ ‐ *A*_λ700_)_pH=4.5_

*D*_f_: volume of diluted samples; *L*: cuvet length (1 cm)

*M*_w_: molecular weight cyanidin‐3‐glucoside 449.2 g/mol; *ɛ* = 26,900 L/mol

##### Encapsulation efficiency

The encapsulation efficiency was conducted with two steps. The first stage aimed to extract the anthocyanin compounds which were not microencapsulated, and the second stage consisted of the rupture of the microparticles, to quantify the anthocyanin compounds contained therein. The encapsulation efficiency was calculated via the following equation (García‐Conesa et al., 2018):

$$\text{Encapsulation efficiency}\;\left(\%\right)=\frac{\mathrm{TAC}-\mathrm{SAC}}{\mathrm{TAC}}\times 100$$

TAC: Total anthocyanin content of particles after rupture (mg/g).
SAC: Total anthocyanin content on the surface of particles (mg/g).

#### 2.2.10 Anthocyanin color evaluation

The color of the spray‐dried anthocyanin powder was measured by using colorimeter with a CIELab scale (L*, a*, and b*). The L* means brightness(light–dark), and the larger value is brighter. The a* means red‐green components, a positive value means reddish and a negative one is greenish, and the b* means yellow‐blue components. Color intensity in terms of chroma (C*) was calculated by the following formula:

$$C^{*}=\sqrt{a^{*2}+b^{*2}};h^{*}={\frac{1}{\tan}}^{.}\frac{b^{*}}{a^{*}}$$

### 2.3 Statistical analysis

The experiments were conducted in triplicate and the data were reported as mean ± standard deviation. The results of ANOVA analysis, comparison of the differences between treatments through LSD test, and optimization were processed by JMP version 10.0 software. Microsoft Excel 2016, IBM SPSS Statistics 20, and JMP 10.0 software were used for Statistical analysis.

## 3 RESULTS AND DISCUSSION

### 3.1 Spray‐drying of extracted anthocyanin

#### 3.1.1 Effects of carrier agents, inlet temperature, and flow rate

**FIGURE 1:**
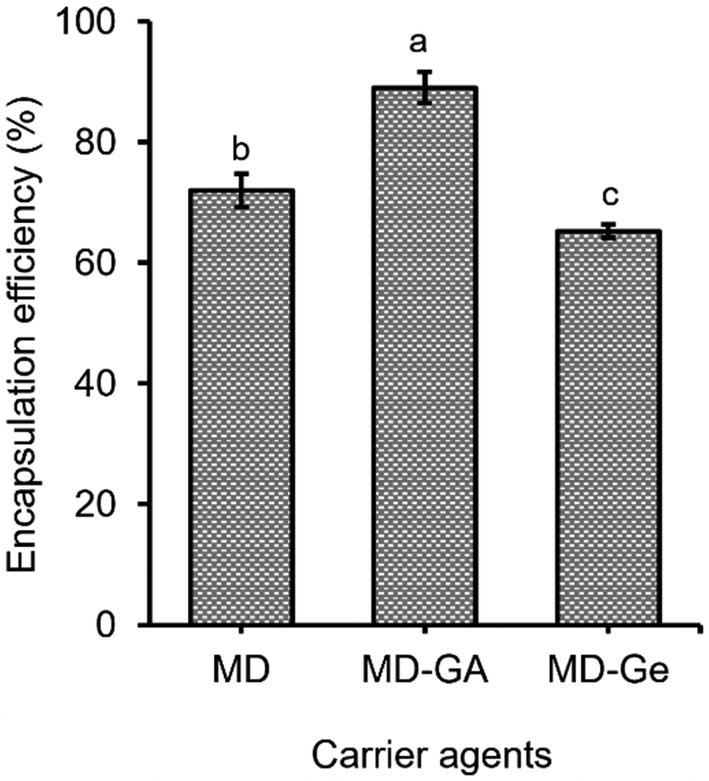
Effects of different types of carrier agents on antshocyanin encapsulation efficiency. *Note*: ^a–c^Different letters on top of bars indicate significant differences (*p* < .05).

Spray‐drying is used to obtain anthocyanin powder from its extract for further convenient utilities. Carriers are an important component that impacts the efficiency of the spraying–drying process because they help to form the polymer matrices to enhance the encapsulation efficiency, which may impair the retention properties and crust‐forming capacity. In this study, carriers included maltodextrin (MD), maltodextrin–gelatin (MD‐Ge), and maltodextrin–gum arabic (MD‐GA) were investigated. The dry matter content of anthocyanin extract after vacuum concentration was around 18%Bx, it was adjusted to 25%Bx. The other fixed parameters were the temperature of 140°C, the input flow rate of 7 mL/min, and the carriers of MD, MD–Ge (1:1, w/w), and MD–GA (1:1, w/w), respectively. From the results, the carrier of maltodextrin and gum arabic gave a higher anthocyanin encapsulation efficiency (89.06%) compared to the others (Figure 1).

In fact, a carrier for encapsulation with essential properties such as carbohydrates, proteins, and polysaccharides lead to high efficiency as gum arabic is a typical example due to its water‐soluble, fat‐insoluble, and low viscosity. Arabic is very stable in an acidic medium, so it is good for stabilizing the aroma of fruit juices. The natural pH of the arabic solution (3.9–4.9) is due to the presence of gluconic acid, which is similar to the pH value of anthocyanin extract from *H. sabdariffa* L. Hence, it helps to increase recovery efficiency (Gharsallaoui et al., 2007). In addition, anthocyanins and flavonols can form complexes with polysaccharides, so gum arabic combined with maltodextrin could give higher effectiveness. Maltodextrin lacks the capacity of emulsification and filming while gelatin can form a film but is less stable in acids than gum arabic (Sajilata & Singhal, 2005). Thus, MD or MD–Ge carriers also resulted in lower effectiveness than MD–GA. The high efficiency of MD–GA was also demonstrated by encapsulating anthocyanin from grapes and roselle (Burin et al., 2011; Idham et al., 2012).

**FIGURE 2:**
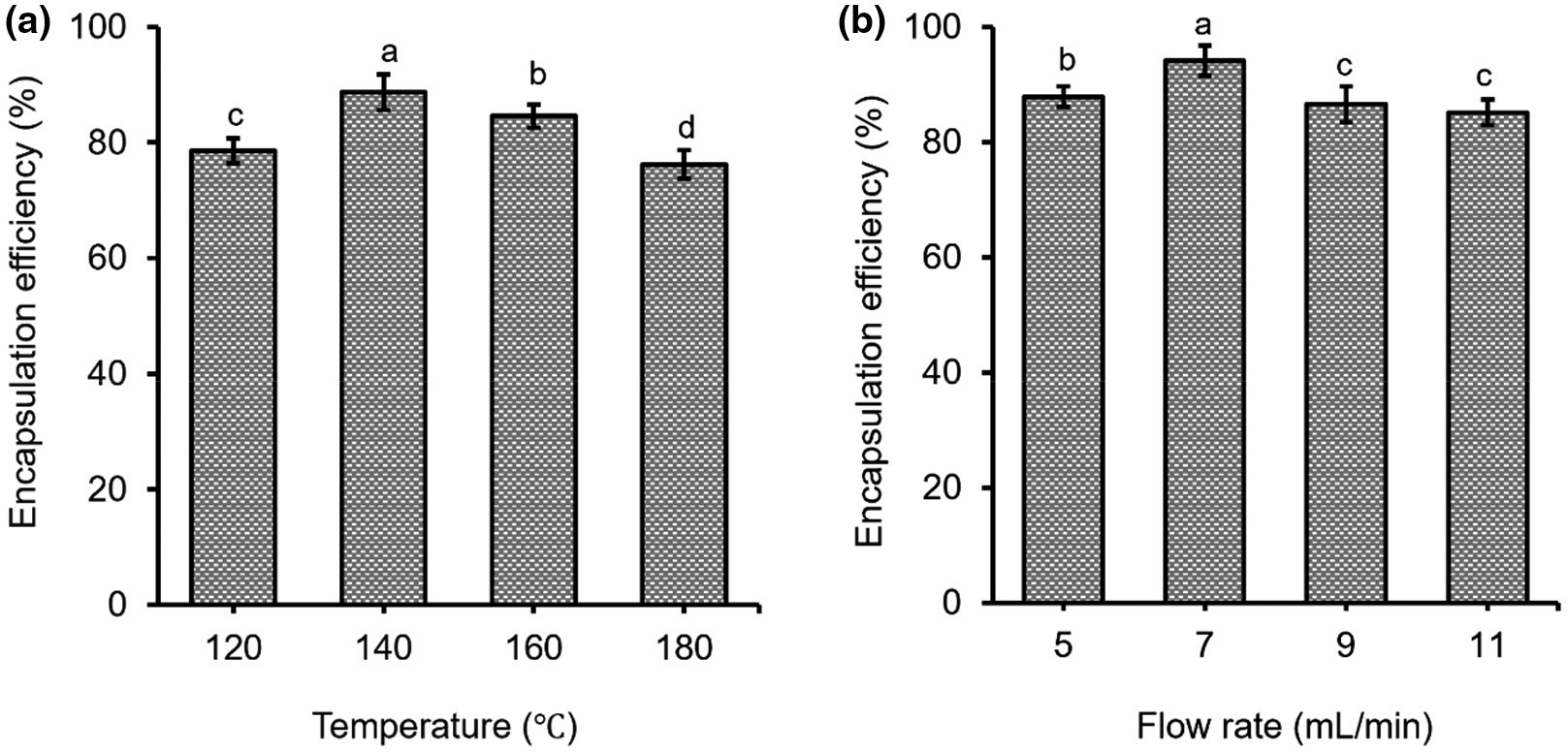
Effects of temperature (a) and flow rate (b) on encapsulation efficiency of anthocyanin‐rich powder in the spray‐drying process. *Note*: ^a–d^Different letters on top of bars indicate significant differences (*p* < .05).

Temperature affects the encapsulation efficiency of the spray‐drying process. It is obvious that microcapsules with different sizes result from the inlet temperature in the spray dryer. In fact, an increase in inlet temperature is related to the acceleration of the rate of drying of the droplets. The high rate of heating could make microcapsules with cracks, breaks, and contractions, which rapidly crust expanded (Mahdi et al., 2020). The low diffusion rate of water molecules is seen at low inlet temperatures of the spray‐drying which leads to a longer process and causes shrinkage and collapse of structures. Smaller capsules with deep surface dents might result from the solidification of the wall materials before the onset of expansion (Ozdemir et al., 2021). The efficiency increased from 78.61% (120°C) to 88.74% (140°C) and decreased to 76.22% at higher inlet temperature (180°C; Figure 2a). In our study, 140°C was chosen for the inlet temperature.

The flow rate can impact on drying process in cases of problems such as clogging of the spray nozzle. The feed flow should be adjusted for the liquid that evaporates before contacting the drying chamber walls. The feed flow increase would result in larger droplet sizes and lower residence time, which lowers the contact time between the product and the high air temperature, preserving bioactive compounds. By contrast, higher feed flow rates could cause an undesirable that the powder contains higher water content because the dried material did not expose to hot air well. So, the moisture of the powder was maintained at a high level which is caused by particles sticking on the machine chamber (Murugesan & Orsat, 2012). In this study, the investigated input flow rate ranged from 5, 7, 9, and 11 mL/min. The flow rate from 5 to 7 mL/min, and the encapsulation efficiency increased from 87.89% to 94.11%. However, the encapsulation efficiency decreased to 85.15% (11 mL/min; Figure 2b). Thus, an inlet flow rate of 7 mL/min was selected in this experiment.

#### 3.1.2 Optimizing spraying–drying conditions

After the above single‐factor experiments, RSM was used to optimize the process to have the powder with high total anthocyanin content. Two factors of temperature and flow rate were designed by the CCD model with the responses of total anthocyanin content, and yield recovery (Table 1).

**TABLE 1:** Full experimental design results according to the CCD model.

No.	Coded variables		Uncoded variables		Encapsulation efficiency (%)
	*X*_1_ (°C)	*X*_2_ (mL/min)	*X*_1_ (°C)	*X*_2_ (mL/min)	
1	−1	−1	120	5	84.12
2	−1	1	120	9	75.97
3	1	−1	160	5	85.33
4	1	1	160	9	90.55
5	−1.41	0	111.8	7	82.41
6	1.41	0	168.2	7	85.78
7	0	−1.41	140	6.18	92.11
8	0	1.41	140	9.82	90.01
9	0	0	140	7	96.22
10	0	0	140	7	94.96
11	0	0	140	7	95.06
12	0	0	140	7	92.81

*Note*: The ANOVA analysis of the quadratic model was evaluated via the values of *F*, *p*, and *R* ^2^ (Table 2).

**TABLE 2:** Regression analysis of spray drying anthocyanin.

Source	df	SS	MS	*F* value	Prob > *F*
Model	5	365.29	73.06	9.33	0.0085*
Error	6	46.96	7.83		
C. Total	11	412.25			
Lack of fit	3	40.90	13.63	6.74	
Pure error	3	6.06	2.02		
Total error	6	46.96			

*Note*: Coefficient of determination, *R* ^2^ = 0.89, adjusted *R* ^2^ = 0.79, *significant at *p* < 0.01.

The *F*‐values were 6.74, satisfied *p* < 0.05, proving that the lack of conformity of the model is negligible. The *p* values of models were lower than .05 and values of Lack of Fit were higher than .05. The coefficients with *p* values >0.05 were eliminated from the models. From linear regression analyses of 12 experiments (using JMP software, version 10.0), a quadratic regression equation of anthocyanin content (*Y*) is as follows:

(5) $$Y=94.76+2.57X_{1}–6.23\;X_{1}X_{1}–2.75\;X_{2}X_{2}$$

**FIGURE 3:**
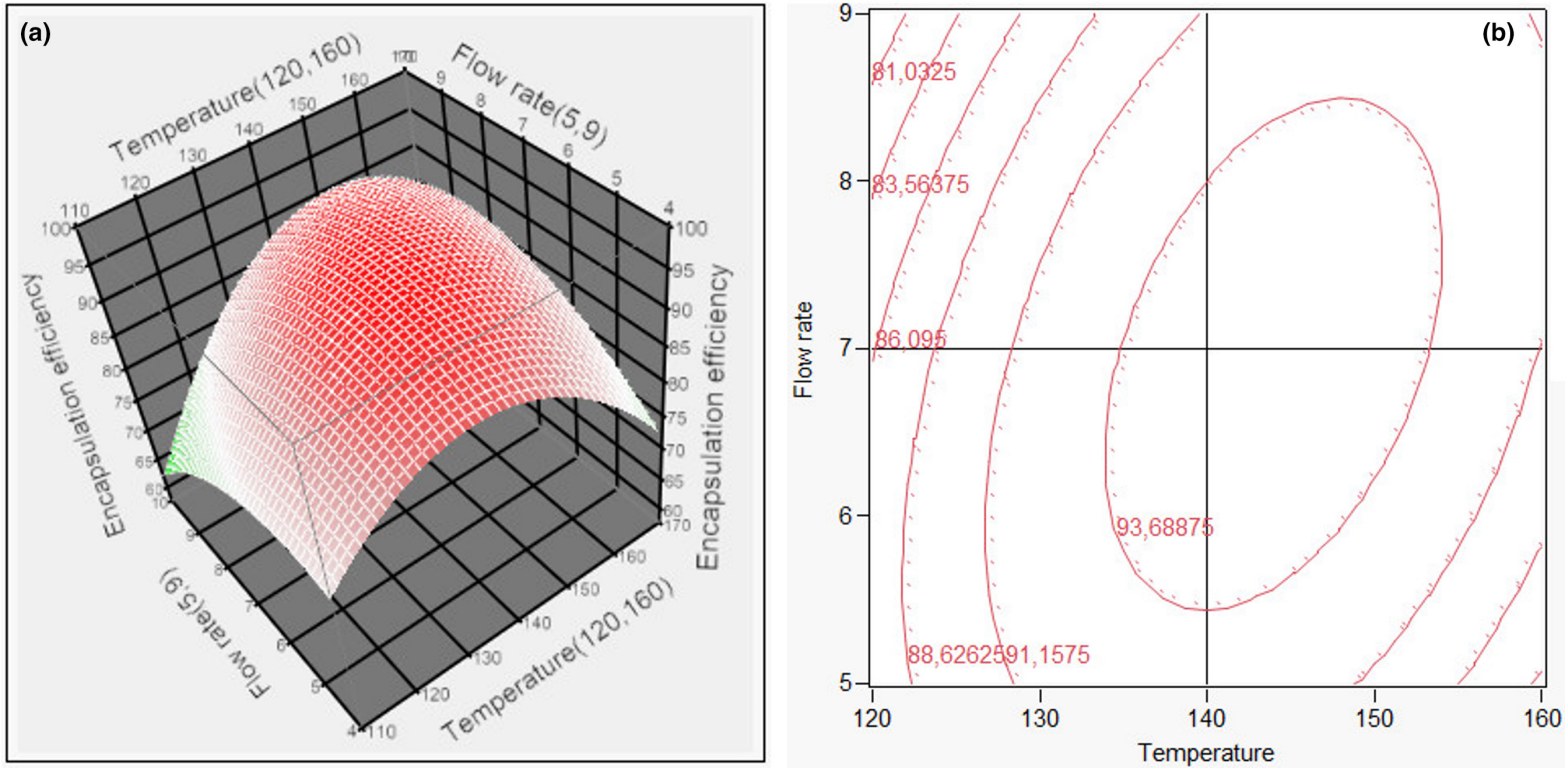
Response surface (a) and contour plots (b) model indicated the influence of factors (Temperature, *X*_1_; Flow, *X*_2_) on the encapsulation efficiency.

According to the regression model, linear coefficient (b_1_) showed a positive impact, and coefficients (b_11_ and b_22_) resulted in negative effects. Besides, the index of *R* ^2^ and *R* ^2^_Adj_ for encapsulation efficiency was (0.89 > 0.8 and 0.79, respectively). Hence, these quadsratic models were appropriate for estimating recovery yield and anthocyanin content in spray‐drying which were observed by RSM with CCD design. From the above results, the two given quadratic models were appropriate for estimating recovery yield and anthocyanin content in this experimental condition. The maximal value of the encapsulation efficiency predicted by the models and the optimal conditions was 95.03% at the optimal conditions of 144.07°C and flow rate of 6.97 mL/min. Three practical experiments at 144°C and a flow rate of 7 mL/min, resulted in powder with anthocyanin content of 100.22 mg/g and the encapsulation efficiency of 93.87%. There was no significant difference between predicted and experimental efficiency (<5%). Besides, the counterplots of response surfaces for spray drying were shown in Figure 3. In short, 144°C and 7 mL/min were the optimal conditions for spray‐drying to obtain anthocyanin powder.

### 3.2 Characterization of anthocyanin powder

The obtained anthocyanin powder was determined by physicochemical properties such as moisture, bulk density, particle density, porosity, flowability, solubility, and encapsulation efficiency. The results are shown in Table 3.

**TABLE 3:** Physicochemical properties of spray‐dried anthocyanin powder.

No.	Criteria	Unit	Results
1	Moisture	%	5.14 ± 0.14
2	Bulk density	g/cm^3^	0.32 ± 0.52
3	Particle density	g/cm^3^	1.81 ± 0.39
4	Tapped density	g/cm^3^	0.52 ± 0.42
5	Flowability	CI (%)	38.17 ± 2.96
		HR	1.64 ± 0.24
6	Porosity	%	70.26 ± 0.37
7	Solubility	%	90.52 ± 3.96
8	Encapsulation efficiency	%	95.47 ± 1.98

The moisture of anthocyanin powder was 5.14%, which meets the moisture requirements for food powder (4%–6%) for long‐term storage. Besides, bulk and tapped density are essential elements related to transport cost, packaging considerations, and color of the powder product. They also link the moisture content, particle size, and particle shape. The tapped density is the actual solid density and does not account for spaces between particles, unlike the apparent density, which accounts for all these spaces (Mahdi et al., 2019). In the current study, the bulk density was quite high (0.32 g/cm^3^), and the particle density and tapped density were 1.81 and 0.52 g/cm^3^, respectively. The bulk density was quite high (0.32 g/cm^3^), ranging from 0.28 to 0.40 g/cm^3^, demonstrating low air content in the powder that can help prevent oxidation during storage (Carneiro et al., 2013). According to Ferrari et al., maltodextrin and gum arabic used as carrier agents resulted in higher bulk density values of spray‐dried blackberry powder. It is easier for heavier material to accommodate spaces between the particles, occupying less space and resulting in higher bulk density values (Ferrari et al., 2012).

Porosity is an important property of powdery materials in the food industry. This is especially crucial in reverting the spray‐drying powder. The porosity of the powder indicates the ratio of gaps between particles and the volume of spaces to the total volume occupied by the powder. The higher values of porosity mean a larger number of spaces between the particles, which may contain oxygen that can cause oxidation reactions. The porosity of the anthocyanin powder was 70.26%, this result was consistent with the results in the previous study with 68%–75% in the porosity (Tonon et al., 2010).

The Carr index and Hausner ratio have a close relation, allowing assessment of the flowability of a powder based on bulk density and particle density (Saker et al., 2019). The CI and HR values of anthocyanin in the current study were in order 38.17%, indicating poor flowability, and 1.64, representing high powder adhesion. In other words, the obtained anthocyanin powder was poor flowability but easy compression. These results were similar to the values of CI (33.72%–48.65%) and HR (1.53–1.96) of spray‐drying powder from flax oil and zein in the study of Quispe‐Condori et al. (2011).

Solubility is an essential element to determine the quality of the powder that is used in the food industry. Poorly soluble powders can result in difficult processing treatment and economic losses. The result is that the solubility of the anthocyanin powder was 90.52% in around 20 s, which is higher than the study of Du et al. (2014) of spray‐dried powder of persimmon pulp (Du et al., 2014).

The encapsulation efficiency is a key indicator in both the microencapsulated process and reverting the spray‐dried one. The effectiveness of microencapsulation was evaluated based on the particles inside the microcapsule compared to the total ones. The result in this study was 95.47% which was in line with the report of Idham et al. (2012) who used only maltodextrin in spray‐drying anthocyanins from *H. sabdariffa* L.

#### 3.2.1 Scanning electron microscopy

**FIGURE 4:**
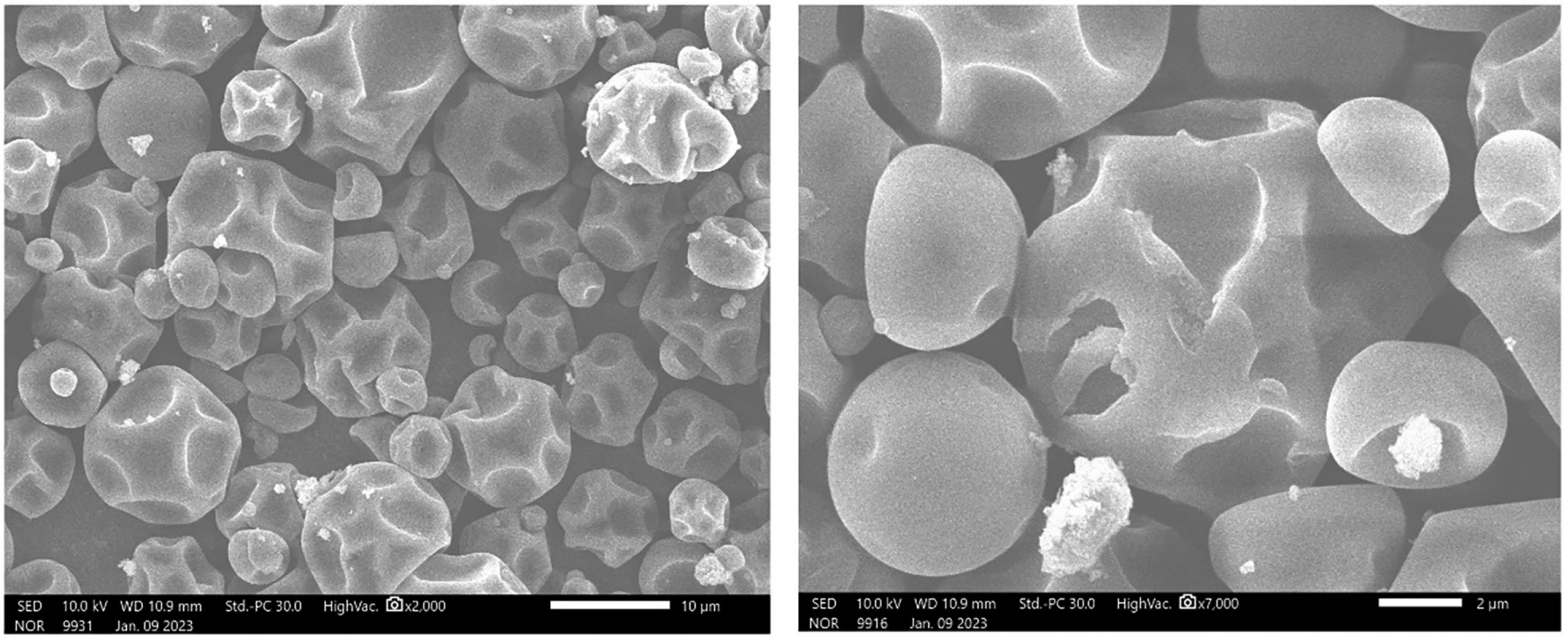
Micrographs of particles of the anthocyanin powder, SEM at ×2000 (left) and at ×7000 (right).

The surface microstructure of the anthocyanin powder via SEM analysis was recorded at a scale of 2 μm and 10 μm (Figure 4). These particles, most of the time, were low particle size (2–10 μm) with a wrinkled, rough surface. Their shape was spherical, unequal in size, with indentations and slight cracks on particles. The different shapes and sizes of particles are associated with agglomeration between smaller particles along with the presence of maltodextrin (Alamilla‐Beltran et al., 2005). The spray‐drying process helps to reduce the pressure on the particle surface due to rapid transpiration, leading to the formation of wrinkles and indentations on particle surfaces (Carneiro et al., 2013). However, the particle surface with cracks could create a diffusion resistance structure in the powder as well as mechanical stress was desirable for the powder products. In addition, the appearance of cracking proves that the microencapsulated process is successfully performed (Guadarrama‐Lezama et al., 2012).

#### 3.2.2 X‐ray diffraction analysis (XRD)

**FIGURE 5:**
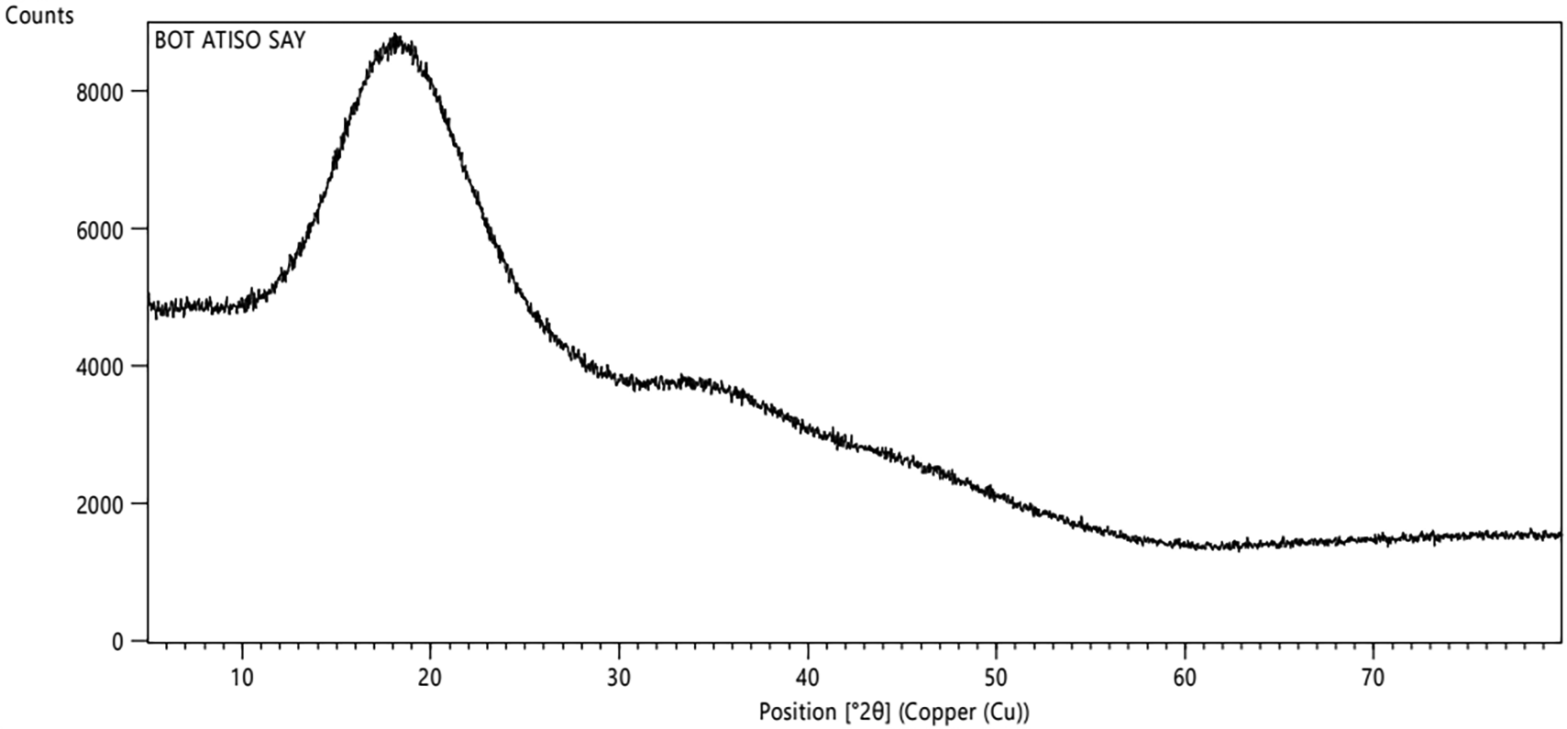
X‐ray diffraction of the anthocyanin powder.

The crystalline state is welcomed for the stability of powder used in food industries, which can be determined by XRD analysis. XRD analysis helps determine the crystalline or amorphous structure of microcapsules in powder samples, thereby assessing crystallinity as related to their stability. For the amorphous state, the molecules are displayed in a disorderly way, creating dispersed bands, whereas the crystalline materials produce sharp and defined peaks due to a highly ordered state (Kang et al., 2019). The XRD schema of the anthocyanin powder was shown in Figure 5.

The XRD result showed that the sample had very low crystallinity and diffuse wide peaks, which indicated the amorphous state of the sample. Amorphous states tend to absorb moisture and water during storage to form agglomerations (Nambiar et al., 2017) which become more sticky and have negative effects because water absorption is associated with weight gain. Products with an amorphous structure are considered more soluble than crystalline forms. Due to the amorphous structure of maltodextrin and the powder without clear peaks, it indicates that microcapsules do not form other phases such as particles with individual crystalline structures but still exist inside maltodextrin particles (Figure 5). This result was also similar to the XRD scheme of an amorphous structure of spray‐drying powder from *Urtica dioica* L. extract in the study by Kalajahi and Ghandiha (2022).

#### 3.2.3 FT‐IR analysis

**FIGURE 6:**
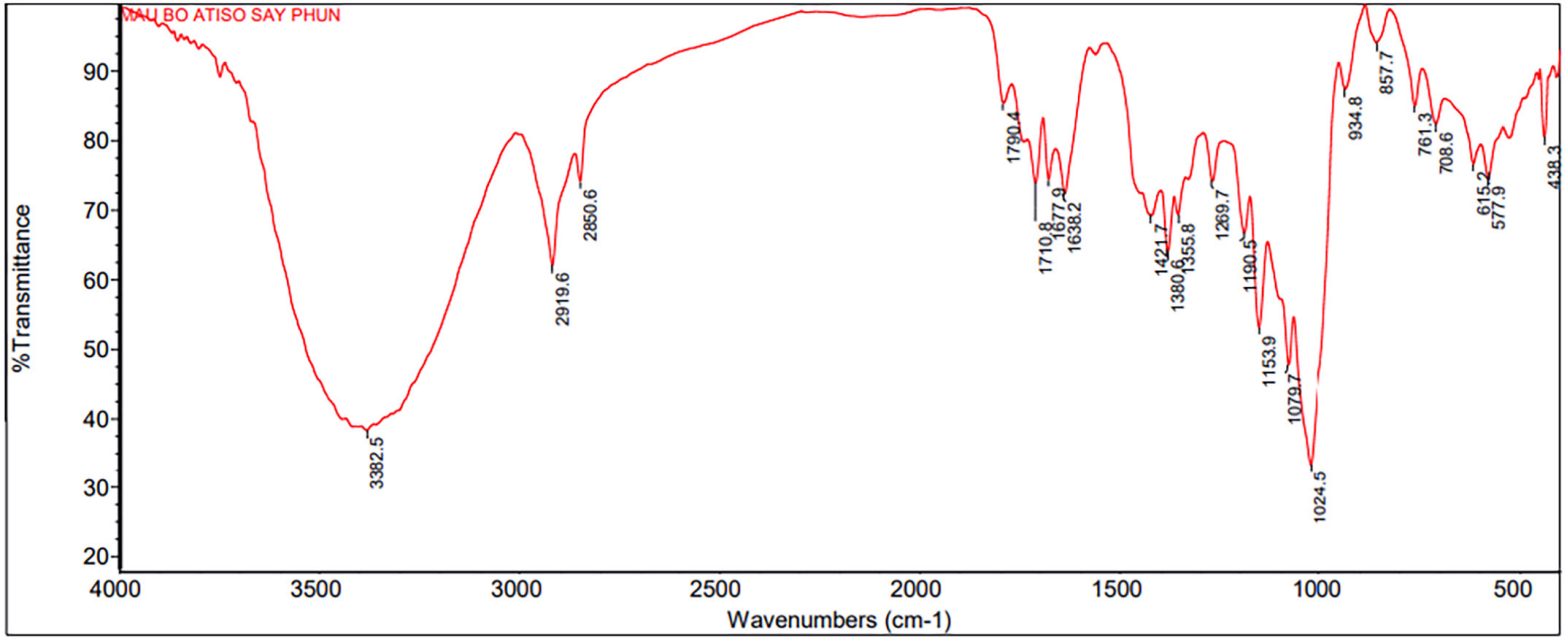
FT‐IR spectra of the anthocyanin powder.

The functional groups and the nature of molecular interactions were shown by using FT‐IR spectroscopy. The FT‐IR spectrum of the anthocyanin powder sample was given in Figure 6.

It can be observed that the characteristic bands in the FT‐IR spectrum of the bands at 3382.5/cm, which could be attributed to the stretching vibration of the hydroxyl group of alcohols or phenols. The peaks at 2919.6 and 2850.6/cm were attributed to ‐C‐H stretching. The wavenumber length of 1710.8 and 1638.2/cm could be assigned for C=O and C=C stretching in anthocyanins. The characteristic peak at 1024.5/cm with a strong signal could be attributed to the C–H deformation of the aromatic rings (Kang et al., 2019; Teixeira et al., 2022).

### 3.3 Apply anthocyanin powder to marshmallows

#### 3.3.1 Marshmallow production and sensory evaluation

**FIGURE 7:**
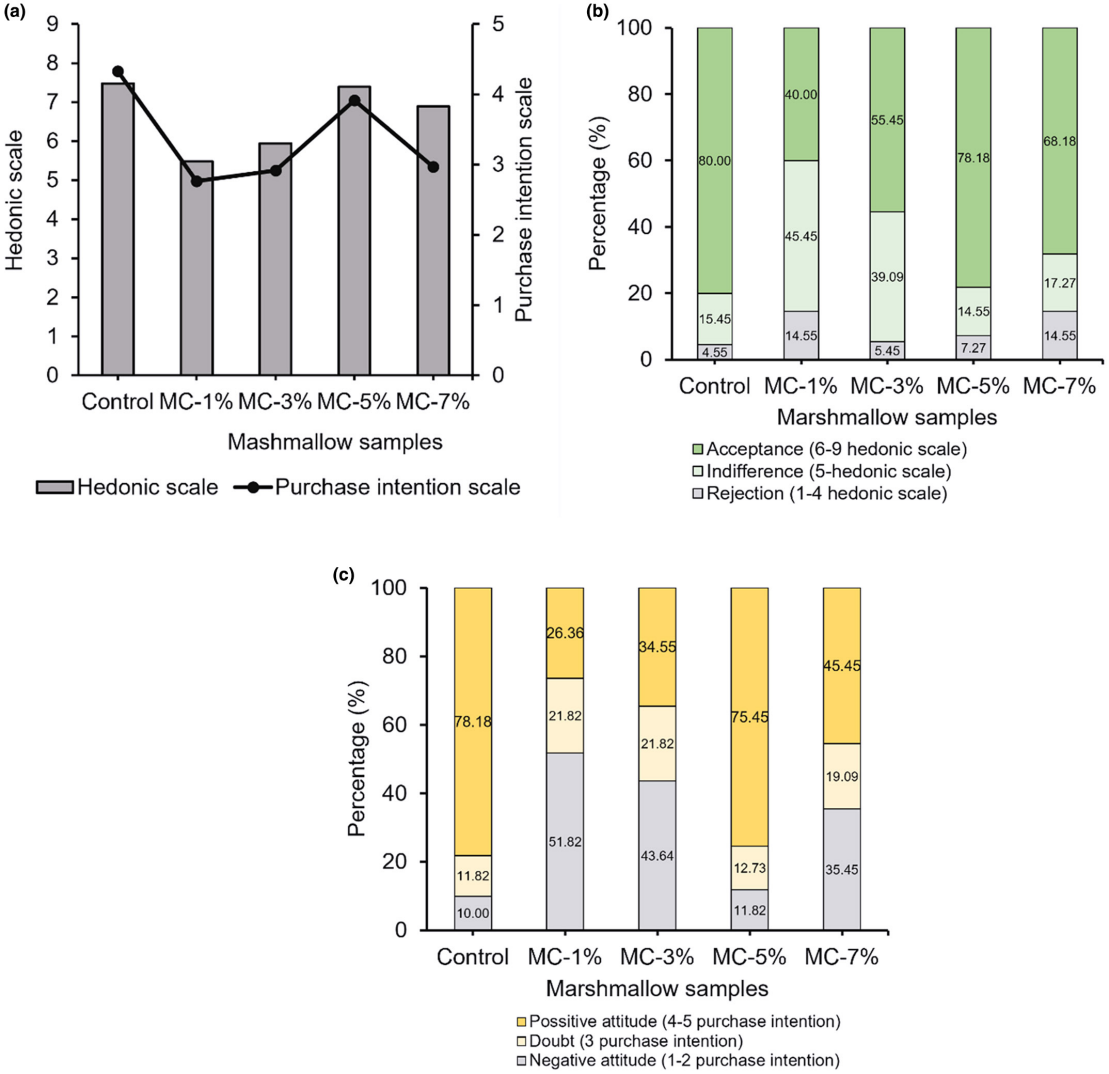
The hedonic scale of marshmallow samples loaded with anthocyanin powder (a), the acceptability of the candy samples (b), and the scale of the intention of purchase (c).

The amount of anthocyanins added to food is unlimited. In this study, anthocyanin powder was added 1%–7% d.m. by weight into marshmallows. The amount of added anthocyanin powder would affect the color and taste of the product, and the marshmallows were evaluated via a sensory test to choose the appropriate percentage rate. In acceptable sensory tests, a group of 110 testers of marshmallows loaded with anthocyanin, of those 49 were male and 61 were female. The results were shown in Figure 7a.

For the acceptability of appearance according to the hedonic scale, all the samples were different from each other (*p* ≤ .05), with the control sample (marshmallow with synthetic color) and MC‐5% (marshmallow containing 5% anthocyanin powder) as the most accepted all, with mean values (7.48 and 7.39, respectively) corresponding to between “like moderately” and “like very much.” The MC‐7% sample was considered close to “like moderately” with a mean of 6.89. The samples of MC‐1% and MC‐3% with mean values in order of 5.48 and 5.95 referred to “either like or dislike” and “like slightly.” The essential element for the acceptability of MC‐5% candy appearance color and smell homogeneity. Besides, samples MC‐1% and MC‐3% were slightly pale while the MC‐7% sample had a deep pinkish color and a carrot‐like taste, which was less desirable to the testers.

Concerning acceptance, MC‐5% and the control samples were highly accepted, at 80%. By contrast, MC‐1% and MC‐3% samples were the lowest acceptance with 40% and 55%, respectively. Although MC‐7% sample acceptance was 68.18%, its rejection was 14.55% (Figure 7b).

Turning to the purchase intention (Figure 7c), 78.18% of consumers have had a positive attitude toward purchasing the MC‐5% and 75.45% for the control sample, which suggests “probably would buy it” in the scale used. As to purchase intention for the MC‐7% sample, 45.45% of consumers indicated positive intention, and the figures for negative and bout intention were 34.55% and 26.36%. The number of customers who owned their negative intention for MC‐1% and MC‐3% outweigh the positive ones.

#### 3.3.2 Characterization of marshmallow

The marshmallow MC‐5% was characterized in terms of physical–chemical, micro‐bacterial, and nutritional factors (Table 4).

**TABLE 4:** Characterization of marshmallow containing 5% anthocyanin powder.

No.	Criteria	Unit	Results	No.	Criteria	Unit	Results
1	Moisture	%	24.23 ± 0.17	9	Ash insoluble in hydrochloric acid	%	0.06 ± 0.01
2	pH		3.55 ± 0.02	10	Total carbohydrate	%	74.45
3	L*		43.89 ± 1.28	11	Reducing sugar	%	32.12
4	a*		31.63 ± 1.07	12	Total aerobic microorganisms	CFU/g	1.2 × 10^3^
5	b*		12.67 ± 1.03	13	*Coliforms*	CFU/g	Not detected
6	C*		35.07 ± 0.98	14	*Escherichia coli*	CFU/g	Not detected
7	Protein	%	0.27	15	*Clostridium perfringens*	CFU/g	Not detected
8	Lipid	%	0.12	16	Total number of yeast and mold spores	CFU/g	Not detected

According to Table 4, marshmallows had an attractive red color (L* = 43.89, C* = 35.07). The physicochemical, and microbiological parameters, nutritional ingredients, and the total sugar content of MC‐5% sample met the requirements of TCVN 5908–2009 national regulations on confectionery products. The anthocyanin content and antioxidant activity of the selected marshmallow were determined after 0, 14, and 30 days. The results are shown in Table 5.

**TABLE 5:** Anthocyanin content and DPPH scavenging of MC‐5% marshmallow after 0, 14, and 30 days.

Time (days)	Total anthocyanin (m/g)	DPPH scavenging (%)
0	4.89 ± 0.102^a^	55.16 ± 2.122^a^
14	4.62 ± 0.09^b^	40.13 ± 1.894^b^
30	4.18 ± 0.08^c^	28.17 ± 1.073^c^

*Note*: Different letters in the column indicate significant differences (*p* < 0.05).

The content of anthocyanin in marshmallows decreased significantly during the surveyed time from 4.89 to 4.18 mg/g after a month. Regarding the antioxidant activity of marshmallows in storage, the DPPH scavenging capacity decreased gradually from the initial day (55.16%) to 14th day (40.13%) and 30th day (28.17%). The ability of anthocyanin for free radical scavenging decreased when added to candy during storage time, possibly due to their interaction with metal ions and other components in the candy and reduced mobility of the OH group leading to reduced free radical scavenging capacity. Anthocyanin proves its natural pigment with potential antioxidant activity together with its water‐soluble property. That is the reason why it is widely used in the food industry as an alternative to synthetic colorants.

## 4 CONCLUSION

The study found the optimal spray‐drying conditions (144°C and 7 mL/min) to obtain the anthocyanin powder from *H. sabdariffa* L. extract with carrier agents of GA and MD. The pigment powder that was encapsulated by spray drying contained 100.22 mg/g anthocyanin with the encapsulation efficiency of 93.87%. The product had acceptable hygroscopicity, high water solubility, poor flowability, easy compression, and good encapsulation efficiency. The SEM analysis, XRD, and FT‐IR spectrum contributed to demonstrating the anthocyanin efficiency in carriers. The initial application of the pigment powder to marshmallows with a 5% addition of anthocyanin had a good appearance on consumers which indicated the potential for food industrial usage as colorants with potential antioxidant activity after a month of storage.

## AUTHOR CONTRIBUTIONS

**Nhon Thi Ngoc Hoang:** Data curation (equal); formal analysis (equal); investigation (equal); methodology (equal); resources (equal); software (equal); visualization (equal); writing – original draft (equal); writing – review and editing (equal). **Nga Ngoc Kieu Nguyen:** Data curation (equal); investigation (equal); software (equal). **Ly Thi Kim Nguyen:** Data curation (equal); formal analysis (equal); investigation (equal); software (equal). **Anh Thi Hong Le:** Conceptualization (equal); formal analysis (equal); investigation (equal); methodology (equal); resources (equal); validation (equal); visualization (equal). **Dao Thi Anh Dong:** Conceptualization (equal); investigation (equal); methodology (equal); project administration (equal); supervision (equal); visualization (equal); writing – review and editing (equal).

## CONFLICT OF INTEREST STATEMENT

The authors declare no conflicts of interest.

## Data Availability

Data available on request from the author.

## References

[fsn33898-bib-0001] Alamilla‐Beltran, L. , Chanona‐Perez, J. J. , Jimenez‐Aparicio, A. R. , & Gutierrez‐Lopez, G. F. (2005). Description of morphological changes of particles along spray drying. Journal of Food Engineering, 67(1–2), 179–184. 10.1016/j.jfoodeng.2004.05.063

[fsn33898-bib-0002] Bertoia, M. L. , Rimm, E. B. , Mukamal, K. J. , Hu, F. B. , Willett, W. C. , & Cassidy, A. (2016). Dietary flavonoid intake and weight maintenance: Three prospective cohorts of 124 086 US men and women followed for up to 24 years. BMJ, 352, i17. 10.1136/bmj.i17 26823518 PMC4730111

[fsn33898-bib-0003] Burin, V. M. , Rossa, P. N. , Ferreira‐Lima, N. E. , Hillmann, M. C. , & Boirdignon‐Luiz, M. T. (2011). Anthocyanins: Optimisation of extraction from cabernet sauvignon grapes, microencapsulation and stability in soft drink. International Journal of Food Science & Technology, 46(1), 186–193. 10.1111/j.1365-2621.2010.02486.x

[fsn33898-bib-0004] Carneiro, H. C. , Tonon, R. V. , Grosso, C. R. , & Hubinger, M. D. (2013). Encapsulation efficiency and oxidative stability of flaxseed oil microencapsulated by spray drying using different combinations of wall materials. Journal of Food Engineering, 115(4), 443–451. 10.1016/j.jfoodeng.2012.03.033

[fsn33898-bib-0005] Da‐Costa‐Rocha, I. , Bonnlaender, B. , Sievers, H. , Pischel, I. , & Heinrich, M. (2014). *Hibiscus sabdariffa* L.–A phytochemical and pharmacological review. Food Chemistry, 165, 424–443. 10.1016/j.foodchem.2014.05.002 25038696

[fsn33898-bib-0006] de Pascual‐Teresa, S. , Moreno, D. A. , & García‐Viguera, C. (2010). Flavanols and anthocyanins in cardiovascular health: A review of current evidence. International Journal of Molecular Sciences, 11(4), 1679–1703. 10.3390/ijms11041679 20480037 PMC2871133

[fsn33898-bib-0007] Du, J. , Ge, Z. Z. , Xu, Z. , Zou, B. , Zhang, Y. , & Li, C. M. (2014). Comparison of the efficiency of five different drying carriers on the spray drying of persimmon pulp powders. Drying Technology, 32(10), 1157–1166. 10.1080/07373937.2014.886259

[fsn33898-bib-0008] Eichhorn, S. , & Winterhalter, P. (2005). Anthocyanins from pigmented potato (*Solanum tuberosum* L.) varieties. Food Research International, 38(8–9), 943–948. 10.1016/j.foodres.2005.03.011

[fsn33898-bib-0009] El‐Messiry, D. M. , El Desoky, S. M. , El‐Razek, A. , & Rabab, H. (2021). Characteristics of children marshmallow candy colored by natural anthocyanin extract from Jamun (*Syzygium cumini*) during cold storage. Journal of Food and Dairy Sciences, 12(8), 189–194. 10.21608/jfds.2021.88671.1024

[fsn33898-bib-0010] Fairlie‐Jones, L. , Davison, K. , Fromentin, E. , & Hill, A. M. (2017). The effect of anthocyanin‐rich foods or extracts on vascular function in adults: A systematic review and meta‐analysis of randomised controlled trials. Nutrients, 9(8), 908. 10.3390/nu9080908 28825651 PMC5579701

[fsn33898-bib-0011] Ferrari, C. C. , Germer, S. P. M. , Alvim, I. D. , Vissotto, F. Z. , & de Aguirre, J. M. (2012). Influence of carrier agents on the physicochemical properties of blackberry powder produced by spray drying. International Journal of Food Science & Technology, 47(6), 1237–1245. 10.1111/j.1365-2621.2012.02964.x

[fsn33898-bib-0012] García‐Conesa, M. T. , Chambers, K. , Combet, E. , Pinto, P. , Garcia‐Aloy, M. , Andrés‐Lacueva, C. , de Pascual‐Teresa, S. , Mena, P. , Konic Ristic, A. , Hollands, W. J. , Kroon, P. A. , Rodríguez‐Mateos, A. , Istas, G. , Kontogiorgis, C. A. , Rai, D. K. , Gibney, E. R. , Morand, C. , Espín, J. C. , & González‐Sarrías, A. (2018). Meta‐analysis of the effects of foods and derived products containing ellagitannins and anthocyanins on cardiometabolic biomarkers: Analysis of factors influencing variability of the individual responses. International Journal of Molecular Sciences, 19(3), 694. 10.3390/ijms19030694 29495642 PMC5877555

[fsn33898-bib-0013] Gharsallaoui, A. , Roudaut, G. , Chambin, O. , Voilley, A. , & Saurel, R. (2007). Applications of spray‐drying in microencapsulation of food ingredients: An overview. Food Research International, 40(9), 1107–1121. 10.1016/j.foodres.2007.07.004

[fsn33898-bib-0014] Guadarrama‐Lezama, A. Y. , Dorantes‐Alvarez, L. , Jaramillo‐Flores, M. E. , Pérez‐Alonso, C. , Niranjan, K. , Gutiérrez‐López, G. F. , & Alamilla‐Beltrán, L. (2012). Preparation and characterization of non‐aqueous extracts from chilli (*Capsicum annuum* L.) and their microencapsulates obtained by spray‐drying. Journal of Food Engineering, 112(1–2), 29–37. 10.1016/j.jfoodeng.2012.03.032

[fsn33898-bib-0015] Idham, Z. , Muhamad, I. I. , & Sarmidi, M. R. (2012). Degradation kinetics and color stability of spray‐dried encapsulated anthocyanins from *Hibiscus sabdariffa* L. Journal of Food Process Engineering, 35(4), 522–542. 10.1111/j.1745-4530.2010.00605.x

[fsn33898-bib-0016] Joshi, D. D. (2012). Herbal drugs and fingerprints: Evidence based herbal drugs. Springer Science & Business Media.

[fsn33898-bib-0017] Kalajahi, S. E. M. , & Ghandiha, S. (2022). Optimization of spray drying parameters for encapsulation of nettle (*Urtica dioica* L.) extract. LWT‐Food Science and Technology, 158, 113149. 10.1016/j.lwt.2022.113149

[fsn33898-bib-0018] Kang, Y. R. , Lee, Y. K. , Kim, Y. J. , & Chang, Y. H. (2019). Characterization and storage stability of chlorophylls microencapsulated in different combination of gum Arabic and maltodextrin. Food Chemistry, 272, 337–346. 10.1016/j.foodchem.2018.08.063 30309553

[fsn33898-bib-0019] Lawless, H. T. , & Heymann, H. (2010). Sensory evaluation of food: Principles and practices (Vol. 2. Springer. 10.1007/978-1-4419-6488-5

[fsn33898-bib-0020] Macz‐Pop, G. A. , Rivas‐Gonzalo, J. C. , Pérez‐Alonso, J. J. , & González‐Paramás, A. M. (2006). Natural occurrence of free anthocyanin aglycones in beans (*Phaseolus vulgaris* L.). Food Chemistry, 94(3), 448–456. 10.1016/j.foodchem.2004.11.038 16417317

[fsn33898-bib-0021] Mahdavi, S. A. , Jafari, S. M. , Assadpoor, E. , & Dehnad, D. (2016). Microencapsulation optimization of natural anthocyanins with maltodextrin, gum Arabic and gelatin. International Journal of Biological Macromolecules, 85, 379–385. 10.1016/j.powtec.2015.01.016 26772915

[fsn33898-bib-0022] Mahdi, A. A. , Mohammed, J. K. , Al‐Ansi, W. , Ghaleb, A. D. , Al‐Maqtari, Q. A. , Ma, M. , Ahmed, M. I. , & Wang, H. (2020). Microencapsulation of fingered citron extract with gum arabic, modified starch, whey protein, and maltodextrin using spray drying. International Journal of Biological Macromolecules, 152, 1125–1134. 10.1016/j.ijbiomac.2019.10.201 31751737

[fsn33898-bib-0023] Mahdi, A. A. , Rashed, M. M. , Al‐Ansi, W. , Ahmed, M. I. , Obadi, M. , Jiang, Q. , Raza, H. , & Wang, H. (2019). Enhancing bio‐recovery of bioactive compounds extracted from *Citrus medica* L. Var. sarcodactylis: Optimization performance of integrated of pulsed‐ultrasonic/microwave technique. Journal of Food Measurement and Characterization, 13, 1661–1673. 10.1007/s11694-019-00083-x

[fsn33898-bib-0024] Maier, T. , Göppert, A. , Kammerer, D. R. , Schieber, A. , & Carle, R. (2008). Optimization of a process for enzyme‐assisted pigment extraction from grape (*Vitis vinifera* L.) pomace. European Food Research and Technology, 227, 267–275. 10.1007/s00217-007-0720-y

[fsn33898-bib-0025] Montes Villanueva, N. D. , & Trindade, M. A. (2010). Estimating sensory shelf life of chocolate and carrot cupcakes using acceptance tests. Journal of Sensory Studies, 25(2), 260–279. 10.1111/j.1745-459X.2009.00256.x

[fsn33898-bib-0026] Murugesan, R. , & Orsat, V. (2012). Spray drying for the production of nutraceutical ingredients—A review. Food and Bioprocess Technology, 5, 3–14. 10.1007/s11947-011-0638-z

[fsn33898-bib-0027] Nambiar, R. B. , Sellamuthu, P. S. , & Perumal, A. B. (2017). Microencapsulation of tender coconut water by spray drying: Effect of *Moringa oleifera* gum, maltodextrin concentrations, and inlet temperature on powder qualities. Food and Bioprocess Technology, 10, 1668–1684. 10.1007/s11947-017-1934-z

[fsn33898-bib-0028] Nhon, H. T. N. , My, N. T. D. , Vi, V. N. T. , Lien, P. T. K. , Minh, N. T. T. , & Dao, D. T. A. (2022). Enhancement of extraction effectiveness and stability of anthocyanin from *Hibiscus sabdari*ffa L. Journal of Agriculture and Food Research, 10, 100408. 10.1016/j.jafr.2022.100408

[fsn33898-bib-0029] Ozdemir, N. , Bayrak, A. , Tat, T. , Altay, F. , Kiralan, M. , & Kurt, A. (2021). Microencapsulation of basil essential oil: Utilization of gum arabic/whey protein isolate/maltodextrin combinations for encapsulation efficiency and in vitro release. Journal of Food Measurement and Characterization, 15, 1865–1876. 10.1007/s11694-020-00771-z

[fsn33898-bib-0030] Quispe‐Condori, S. , Saldaña, M. D. , & Temelli, F. (2011). Microencapsulation of flax oil with zein using spray and freeze drying. LWT‐ Food Science and Technology, 44(9), 1880–1887. 10.1016/j.lwt.2011.01.005

[fsn33898-bib-0031] Saifullah, M. , Yusof, Y. A. , Chin, N. L. , & Aziz, M. G. (2016). Physicochemical and flow properties of fruit powder and their effect on the dissolution of fast dissolving fruit powder tablets. Powder Technology, 301, 396–404. 10.1016/j.powtec.2016.06.035

[fsn33898-bib-0032] Sajilata, M. G. , & Singhal, R. S. (2005). Specialty starches for snack foods. Carbohydrate Polymers, 59(2), 131–151. 10.1016/j.carbpol.2004.08.012

[fsn33898-bib-0033] Saker, A. , Cares‐Pacheco, M. G. , Marchal, P. , & Falk, V. (2019). Powders flowability assessment in granular compaction: What about the consistency of Hausner ratio? Powder Technology, 354, 52–63. 10.1016/j.powtec.2019.05.032

[fsn33898-bib-0034] Santhalakshmy, S. , Bosco, S. J. D. , Francis, S. , & Sabeena, M. (2015). Effect of inlet temperature on physicochemical properties of spray‐dried jamun fruit juice powder. Powder Technology, 274, 37–43. 10.1016/j.powtec.2015.01.016

[fsn33898-bib-0035] Sun, H. , Mu, T. , Xi, L. , Zhang, M. , & Chen, J. (2014). Sweet potato (*Ipomoea batatas* L.) leaves as nutritional and functional foods. Food Chemistry, 156, 380–389. 10.1016/j.foodchem.2014.01.0he79 24629984

[fsn33898-bib-0036] Tan, C. , Xue, J. , Abbas, S. , Feng, B. , Zhang, X. , & Xia, S. (2014). Liposome as a delivery system for carotenoids: Comparative antioxidant activity of carotenoids as measured by ferric reducing antioxidant power, DPPH assay and lipid peroxidation. Journal of Agricultural and Food Chemistry, 62(28), 6726–6735. 10.1021/jf405622f 24745755

[fsn33898-bib-0037] Teixeira, S. C. , de Oliveira, T. V. , Silva, R. R. A. , Ribeiro, A. R. C. , Stringheta, P. C. , Rigolon, T. C. B. , Rigolon, T. C. B. , Stringheta, P. C. , & Soares, N. D. F. F. (2022). Colorimetric indicators of açaí anthocyanin extract in the biodegradable polymer matrix to indicate fresh shrimp. Food Bioscience, 48, 101808. 10.1016/j.fbio.2022.101808

[fsn33898-bib-0038] Tonon, R. V. , Brabet, C. , & Hubinger, M. D. (2010). Anthocyanin stability and antioxidant activity of spray‐dried açai (Euterpe oleracea Mart.) juice produced with different carrier agents. Food Research International, 43(3), 907–914. 10.1016/j.foodres.2009.12.013

